# Achieving Conservation when Opportunity Costs Are High: Optimizing Reserve Design in Alberta's Oil Sands Region

**DOI:** 10.1371/journal.pone.0023254

**Published:** 2011-08-17

**Authors:** Richard R. Schneider, Grant Hauer, Dan Farr, W. L. Adamowicz, Stan Boutin

**Affiliations:** 1 Department of Biological Sciences, University of Alberta, Edmonton, Alberta, Canada; 2 Department of Rural Economy, University of Alberta, Edmonton, Alberta, Canada; University of Western Ontario, Canada

## Abstract

Recent studies have shown that conservation gains can be achieved when the spatial distributions of biological benefits and economic costs are incorporated in the conservation planning process. Using Alberta, Canada, as a case study we apply these techniques in the context of coarse-filter reserve design. Because targets for ecosystem representation and other coarse-filter design elements are difficult to define objectively we use a trade-off analysis to systematically explore the relationship between conservation targets and economic opportunity costs. We use the Marxan conservation planning software to generate reserve designs at each level of conservation target to ensure that our quantification of conservation and economic outcomes represents the optimal allocation of resources in each case. Opportunity cost is most affected by the ecological representation target and this relationship is nonlinear. Although petroleum resources are present throughout most of Alberta, and include highly valuable oil sands deposits, our analysis indicates that over 30% of public lands could be protected while maintaining access to more than 97% of the value of the region's resources. Our case study demonstrates that optimal resource allocation can be usefully employed to support strategic decision making in the context of land-use planning, even when conservation targets are not well defined.

## Introduction

It is becoming widely recognized that trade-offs between conservation objectives and economic objectives need to be addressed as an integral component of the conservation planning process [1], [2], [3]. In recent years, various approaches involving optimal resource allocation techniques have been developed for this purpose [4], [5], [6], [7]. These approaches can help ensure that conservation gains are as great as possible given other land use constraints and that conservation plans do not fail at the point of implementation [2], [8].

Much of the literature on optimal resource allocation involves outcomes defined at the species level. Some applications focus on maintaining viable populations of threatened species [9], [10], [11], [12]. Other applications involve maximizing the number of species protected per dollar invested using a return on investment approach [1], [7]. In both cases the conservation objective can be clearly defined, either in terms of the number of species protected or, in the case of individual species, measures of population viability. This provides a robust basis for constructing an objective function and applying optimization algorithms.

In our study we explore the application of optimal resource allocation to coarse-filter reserve design, in the context of regional land-use planning [13], [14]. Here the objective is to conserve the majority of species within a planning region by protecting a representative array of natural ecosystems and their constituent processes in biological reserves [13], [15]. By protection we mean a prohibition on new industrial development. Thus, the establishment of reserves involves an economic trade-off that can be expressed in terms of the opportunity cost of forgone resource revenues.

The assumption underlying the coarse-filter approach is that the habitat needs of most species will be met if all major ecosystem types in the region are represented. Knowledge of the habitat requirements of individual species is not required, which is what makes this approach workable for conservation at the regional scale. It is understood that a complementary fine-filter approach is required to address needs of species that utilize unique habitat types or have other specialized requirements.

**Figure 1:**
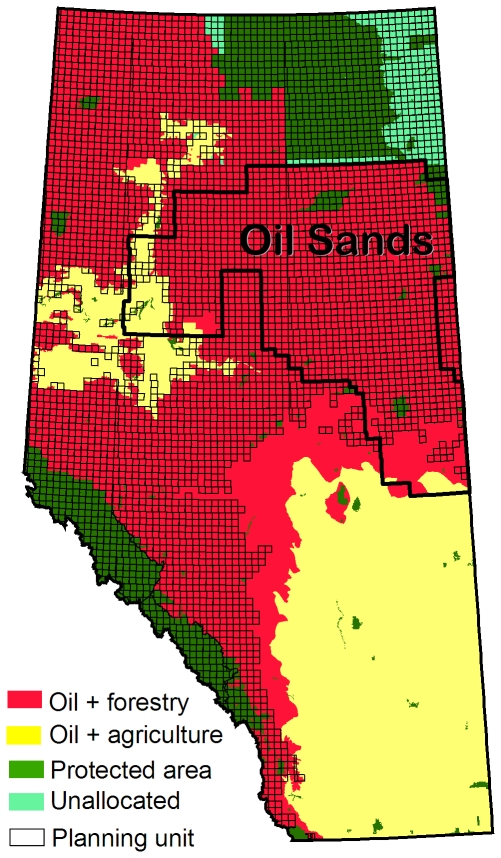
Study area. Planning units (open black rectangles) used in the Marxan study were limited to Alberta townships containing at least 50% public land. Major land-use allocations are also shown (oil refers to both oil and gas extraction). The boundary of the oil sands region is outlined in black.

The setting for our case study is Alberta, Canada. Oil and gas extraction and forestry operations occur across most of Alberta's forested lands (Fig. 1) and there are concerns that the cumulative environmental impacts of these industries are not being adequately addressed [16], [17], [18]. The oil sands, found within a 138,000 km^2^ region of Alberta's boreal forest, are central to these concerns (Fig. 1). Despite the strategic importance of the oil sands to future energy security in both Canada and the United States, the development of this resource has become increasingly controversial. A perceived high level of environmental impact and the very large area of forested land involved has led to local and international demands for a reduction or halt to future oil sands development [19].

Alberta has recently launched a provincial-scale planning initiative, the Alberta Land-use Framework, that aims to achieve a better balance between economic and environmental outcomes through new approaches to land management [20]. One of the proposed approaches involves the establishment of additional biological reserves in which the maintenance of biodiversity and ecological processes are designated as the priority land use [21]. Provincial planning documents list several criteria for the selection of the reserves that collectively are consistent with a coarse-filter approach to reserve design [21].

The challenge in applying the coarse-filter approach is that the relationship between biodiversity outcomes and the degree of ecosystem representation is complex and difficult to quantify [22], [23], [24]. We know that more representation is better, but not how much is enough [25], [26], [27]. Furthermore, ecosystem representation is not the only variable that needs to be considered. For example, design features that influence the integrity and connectivity of reserves also affect biodiversity outcomes, as well as costs [28], [29], [30].

The lack of objectively defined conservation targets complicates the use of optimization techniques in the coarse-filter approach. Knowing the optimal reserve design for an arbitrarily chosen conservation target is of limited value to land-use planners tasked with balancing conflicting societal objectives concerning conservation and economic development. In our study we pursue a hybrid approach that incorporates an analysis of trade-offs in combination with formal optimization. In the trade-off analysis we systematically explore the relationship between conservation targets and economic opportunity costs. We also assess whether these relationships are affected by the scale of planning. We use optimization, via the Marxan conservation planning software, to generate reserve designs for each level of conservation target, ensuring that our quantification of conservation and economic outcomes represents the optimal allocation of resources in each case.

The objective of our study is to characterize and quantify trade-offs associated with the establishment of new reserves on Alberta's public lands. Our findings are intended to help land use planners select a reserve design that provides an optimal balance among competing economic and conservation objectives. More generally, our hope is to advance the adoption of optimization techniques by demonstrating how they can be applied in the context of coarse-filter conservation and regional land-use planning [31], [32].

## Methods

Our study area is comprised of Alberta's public lands (552,240 km^2^; Fig. 1). We excluded the southern agricultural zone from our analysis because only small fragments of native prairie remain, effectively precluding coarse-filter conservation. In the rest of the province public lands are generally forested, and though they have been subject to varying degrees of human disturbance, they still retain most of their natural characteristics. The majority of our study area is comprised of boreal forest; however, mountains and foothills are present in the southwest and a small amount of Canadian shield is present in the northeast (Fig. S1).

For our trade-off analysis we assumed that the conservation objective is to maintain the abundance and distribution of native species through coarse-filter habitat protection and that the economic objective is to maximize economic returns by maintaining resource development opportunities. Although habitat protection is not the only way that biodiversity and economic development can be linked we did not attempt to draw any additional linkages in our study.

### Reserve Design

We used Marxan to quantify the relationship between habitat protection and economic opportunity cost [33]. Marxan calculates solutions (reserve designs) that are optimal in the sense that the spatial configuration of reserves generated by the model achieves the conservation targets at the least economic cost. The design of the reserves was based on five elements: opportunity cost, the amount of protection (in terms of ecosystem representation), intactness, the size of individual reserves, and connectivity among sites. We also considered the scale of planning in our analysis.

#### Opportunity Cost

We define opportunity cost as the value of foregone resource development opportunities resulting from a prohibition on new development within reserves. We expressed this variable as the net present value (NPV) of resources within new reserves as a proportion of the NPV of the total study area.

We determined NPVs for each of the four main industrial sectors active in our study — conventional natural gas, conventional oil, bitumen (a tar-like hydrocarbon found in oil sands), and forest products (Fig. S2) — using models developed by Hauer et al. [34]. These models projected expected resource flows, revenues and costs over time, and opportunity costs of capital in terms of discount or interest rates. From these projections we determined net resource values for each sector in present value terms (i.e., NPV). The true opportunity cost of establishing reserves is less than suggested by our estimates of NPV because industry is subject to various capacity constraints that limit the rate at which resources can actually be extracted. There are also opportunities for spatial substitution of activities. However, using these values in a relative fashion (i.e., expressing opportunity cost as a percent of total NPV) should be instructive for strategic planning.

For the oil and gas models the total amount of recoverable oil or gas available per geological layer in each section of land (∼278 ha) was derived from spatially explicit data on reserves and ultimate potential housed with the Alberta's Energy and Resources Conservation Board and the National Energy Board [35]. The flow of resources over time given successful drilling was derived from estimates published by the Alberta Department of Energy [36]. Seismic, operating costs, and capital costs were also obtained from the Alberta Department of Energy [36]. Drilling costs were derived from Petroleum Services Association of Canada [37]. For the capital intensive oil sands projects, costs and bitumen outputs per well were derived from the Alberta Department of Energy [38], [39]. For each section of land, flows of oil or gas were multiplied by forecasted oil and gas prices, derived from GLJ petroleum consultants Ltd. [40], [41]. This revenue stream was then discounted using a 4% real rate of return on investment. Discounted operating, drilling, and exploration costs were subtracted from this revenue to obtain the expected NPV for each land section.

The NPV of land under forest management accounts for less than 1% of total land resource values but was included for completeness. NPVs for forestry were obtained using the methods described in Hauer et al. [42]. The scheduling of forestry activities was based on maximizing NPV under provincial regulations including sustained yield constraints [42].

#### Ecosystem Representation

We incorporated ecosystem representation using two datasets, representing two distinct scales. The first was the Natural Regions of Alberta, which provides a hierarchical ecosystem classification based on landform, soils, hydrology, climate, and dominant vegetation [43]. There are six Natural Regions and 21 Natural Subregions in the province and we used the Natural Subregions for our analysis (Fig. S1).

For finer-scale ecosystem representation we used vegetation types derived from the Alberta Phase 3 forest inventory, which is based on aerial photography and is maintained by Alberta Sustainable Resource Development. We defined seven vegetation types, reflecting our attempt to define ecologically meaningful units limited by the data available in the Phase 3 inventory: pine, black spruce, white spruce, mixedwood, deciduous, shrub, and peat. Because we were using a coarse-filter approach we did not include forest types that were rare.

#### Intactness

We incorporated intactness on the basis of the density of linear features, summarized by township. Linear features were derived from the Alberta Base Features dataset and included roads, pipelines, and seismic lines (Fig. S3). Given that existing lines will regenerate over time if allowed to do so we felt it would be reasonable to have the model minimize linear feature density, rather than set explicit targets for this feature. This approach allowed us to maximize intactness while keeping the number of model permutations within an acceptable range.

#### Reserve Size (clumping)

The size of individual reserves was an outcome of model runs, not an input. However, we could control the mean size of reserves through a penalty factor applied to the total length of reserve boundaries. As the boundary length penalty is increased, contiguous planning units are increasingly favoured, resulting in clumping of reserves and an increase in their mean size.

#### Connectivity

Major rivers present the only obvious landscape features that might serve as natural corridors in our study area, which is mostly comprised of relatively flat boreal plain. Our exploration of connectivity involved scenarios in which the planning units crossed by one of Alberta's major rivers (Athabasca, Hay, Peace, and North Saskatchewan rivers, and their major tributaries) were forced into the model (Fig. S4).

### Modeling Experiments

To conduct the trade-off analysis we defined a series of modeling scenarios representing different combinations of target levels for the various reserve design elements (Table 1). We used Marxan to generate optimal reserve designs for each scenario and then compared the scenarios in terms of their economic opportunity cost (i.e., the proportion of total NPV contained in the reserve system). In practice, Marxan is run repeatedly for a given scenario to generate a series of “very good” designs because it is not practical to identify the single “best” design. We found that 200 repetitions was sufficient to generate stable mean NPV values, permitting meaningful comparisons to be made among scenarios.

**Table 1:** Conservation design elements and their implementation in Marxan.

Design Elements	Marxan Implementation[1]	Scenario Settings
Ecosystem representation	a) Represent all Natural Subregions	15% to 40% in
	b) Represent all forest types	increments of 5%[2]
Intactness	Minimize linear feature density	Minimize
Size of individual reserves	Promote reserve clumping through a penalty on boundary length	Boundary penalty = 0 or maximal[3]
Connectivity	Include major riparian corridors	Include all or none
Opportunity cost	Minimize NPV of petroleum and forestry resources	Minimize
Scale of planning	Provincial runs vs. independent runs for each planning region	

Notes:

[1] Data sources and maps are provided in Table S1 and supplemental figures.

[2] Numeric targets reflect the percentage of the total area of the feature to be represented in the reserve system.

[3] Maximal is where reserves are as clumped as possible while still achieving all ecological representation targets.

Townships (∼9500 ha) were used as the planning unit in Marxan (n = 5784). Townships within the provincial protected area network were included in every design if 50% or more of the township was protected (Fig. 1). Townships that contained more than 50% private land were excluded from all designs (Fig. 1).

The two ecosystem representation elements were incorporated in model runs as proportional targets. For example, a representation target of 25% meant that at least 25% of each Natural Subregion and 25% of each forest type had to be represented in the final reserve design. Our scenarios included representation targets ranging from 15% to 40%.

NPV and intactness did not have explicit targets, but instead, the model was required to minimize these variables as it worked to achieve the representation targets. We explored the influence of NPV and intactness individually and in combination. When NPV and intactness were both in the model they were weighted equally.

The effect of reserve size was investigated by varying the boundary length penalty from zero to maximal, where maximal was the point at which the reserves were as clumped as they could be while still achieving all representation targets. Connectivity was explored by forcing the model to incorporate all townships along the preselected riparian corridors (Fig. S4).

Although the Alberta Land-use Framework is provincial in scope, the province has been divided into seven regions for the purpose of planning. To determine whether the scale of planning would influence our findings we ran a set of scenarios in which each of province's seven planning regions was modeled independently (Fig. S5). The results of these regional-scale scenarios were compared with comparable scenarios conducted at the provincial scale (the scale at which all other modeling was done).

For visual display of reserve designs we calculated the probability of selection for each planning unit over the 200 repetitions of a given scenario and linked this to a map of Alberta townships. We also mapped the planning units selected in a single run (the best Marxan score), to provide an uncluttered example of what the actual reserve system could look like under a given scenario.

## Results

**Figure 2:**
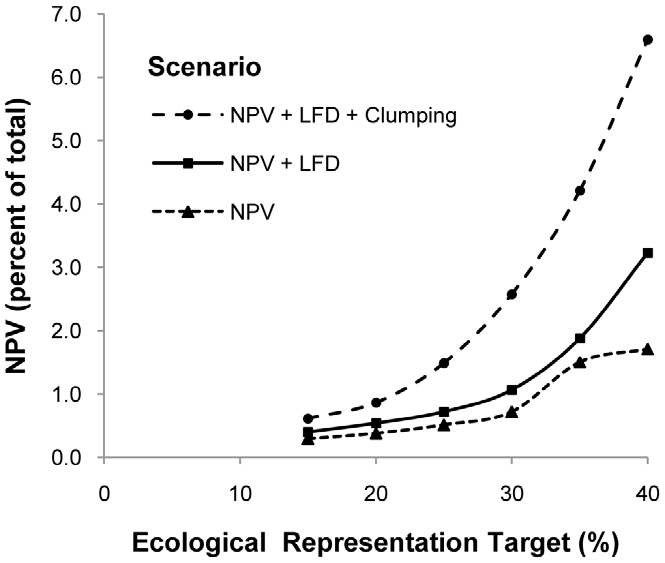
Opportunity cost of the reserve system relative to the ecological representation target. Cost is expressed as a percentage of the NPV of the entire study area. The three lines represent models with different combinations of secondary design variables (see Table 1; LFD = linear feature density).

The relationship between the ecological representation target and opportunity cost (NPV) was nonlinear (Fig. 2). In the scenarios where the model included only the representation target and minimization of cost, the NPV of the reserve system remained less than 1% of the total NPV of the study area until the representation target exceeded 30%. Opportunity costs were more than 20 times higher if the model was not required to minimize cost (for equivalent representation targets and total area of reserves).

Adding the requirement to maximize intactness had minimal effect on opportunity cost except at the 40% representation target (Fig. 2). The effect of clumping on opportunity cost was proportional to the degree of clumping (as set by the boundary length penalty). At maximal levels, clumping resulted in an approximate doubling of opportunity cost across all representation targets (Fig. 2).

**Figure 3:**
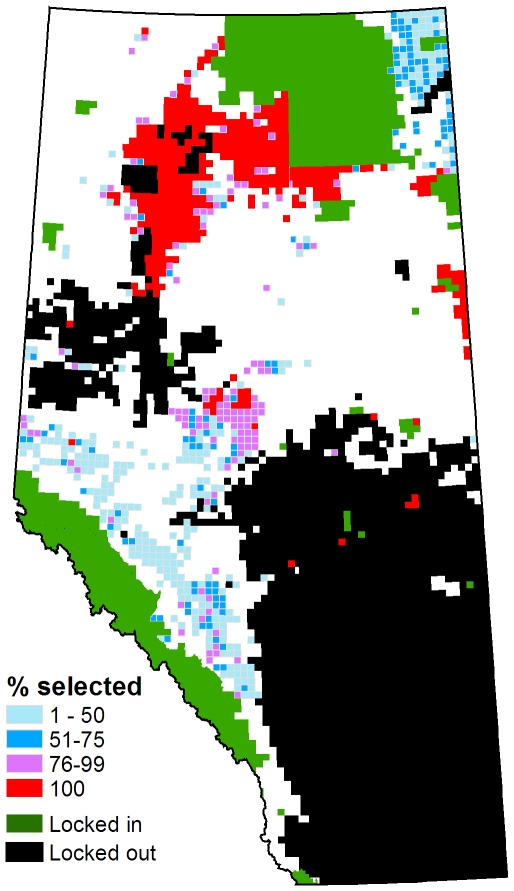
Probability of planning unit selection over 200 Marxan runs. Model includes a 20% ecological representation target and minimization of NPV and linear feature density. There is no penalty on boundary length.

**Figure 4:**
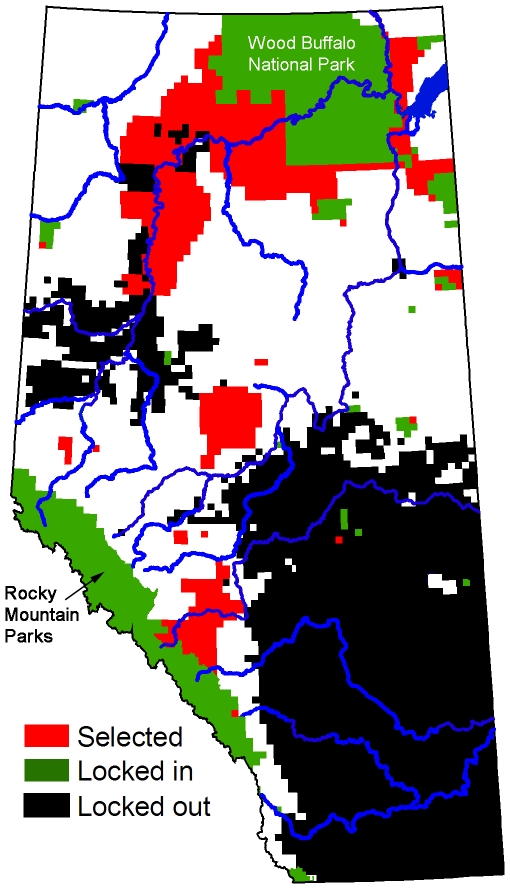
Selected planning units in a single representative run. Model includes a 20% representation target and minimization of NPV and linear feature density. The penalty on boundary length is at its maximum. Major rivers are shown in blue.

For scenarios including ecological representation, intactness and cost, more than half of the planning units in the reserve system were consistently selected each time the model was run (Fig. 3). A substantial degree of aggregation was also evident, even in the absence of the penalty on boundary length. When the boundary penalty was added reserve designs tended to have a consistent spatial pattern involving three large reserves: one centred on Wood Buffalo National Park in northeastern Alberta, a second adjacent to the Rocky Mountain parks in southwestern Alberta, and a third in the Foothills Subregion of west-central Alberta (Fig. 4).

**Figure 5:**
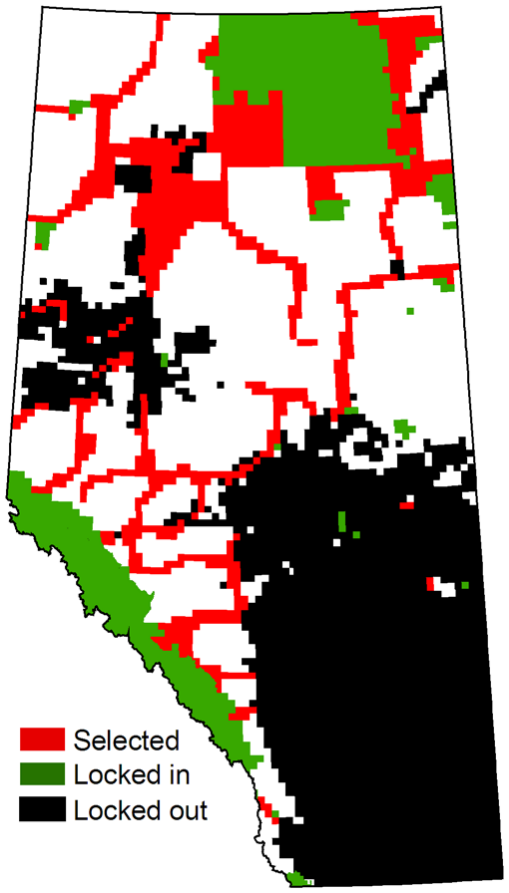
Selected planning units in a single representative run. Model includes a 20% representation target and minimization of NPV and linear feature density. The penalty on boundary length is at its maximum and river corridors are forced into the model.

Corridors based on Alberta's major rivers did not link up in a meaningful way with the spatial distribution of reserves generated by Marxan (Fig. 4). If the river corridors were forced into the model they dominated the reserve designs, resulting in a largely linear reserve system (Fig. 5). Moreover, the opportunity cost of the reserve system increased by more than ten times (with a 20% representation target).

**Figure 6:**
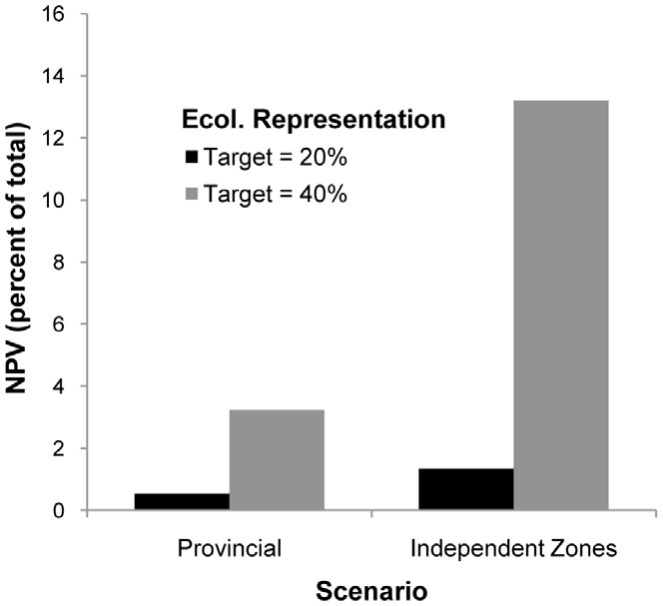
Opportunity cost comparison of provincial vs. regional planning. Cost is expressed as a percentage of the NPV of the entire study area at two levels of representation target.

**Figure 7:**
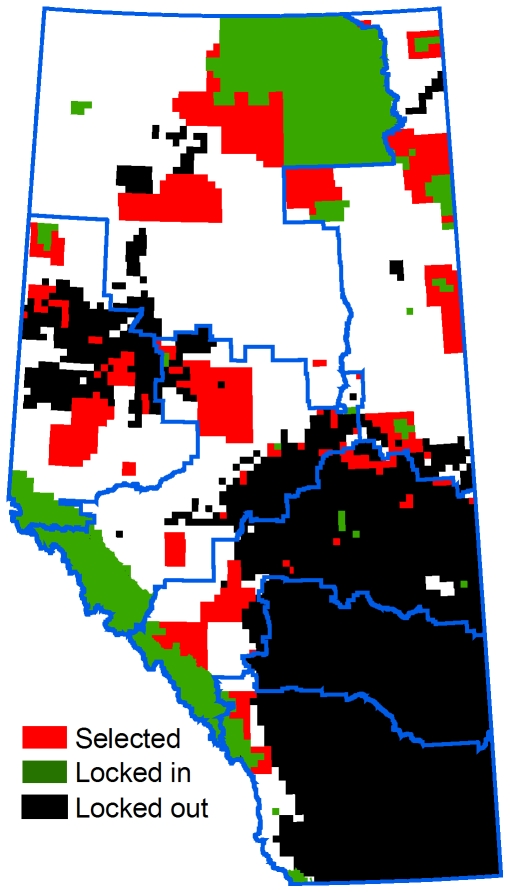
Selected townships in a representative Marxan run using the regional planning approach (i.e., each zone planned separately). The model includes a 20% representation target, minimization of NPV and linear feature density, and maximal penalty on boundary length. Planning regions are outlined in blue.

When conservation planning was conducted independently for each planning region the opportunity cost of the total reserve system was up to 4.1 times higher than for comparable scenarios run at the provincial scale (Fig. 6). The cost differential increased with the level of the representation target (Fig. 6). Regional-scale planning also affected the distribution of reserves. Most notably, much of the Dry Mixedwood Subregion target was achieved adjacent to privately owned agricultural lands instead of relatively intact forests in northwestern Alberta. In addition, the reserves were generally smaller and more widely dispersed (Fig. 7).

## Discussion

The high economic value and wide distribution of Alberta's resources might suggest that little opportunity exists for expansion of the province's system of protected areas. The economic opportunity costs of additional protection are indeed consequential if expressed in terms of the raw value of resources contained within the Marxan reserves. However, if the objective of land-use planning is to achieve a balance between economic and environmental objectives, as it is with the Alberta Land-Use Framework, then raw costs are not a sufficient basis for decision making. A better assessment of the societal trade-offs involved in establishing protected areas can be obtained by expressing the opportunity cost of protection as a percentage of the total value of resources in the planning area. Using this approach we found that the proportion of our study area that is protected could be increased from the current 14.8% to over 30% while maintaining access to more than 97% of the value of the region's resources. It would be hard to argue that this does not represent a reasonable balance from an economic perspective, regardless of what the absolute cost might be.

The reason for this favourable outcome is that the distribution of resource values is highly variable across our study area. Optimization techniques are particularly effective in minimizing the cost of conservation solutions when variance of the cost layer is high [2], [4], [6]. In our case the highly valuable oil sands deposits are responsible for much of the variation in resource values. This being the case, the oil sands could be considered an enabling factor for conservation in Alberta, not a barrier. In practical terms, the establishment of an intensive industry zone in the oil sands region, where economic development is assigned top priority, could generate sufficient economic returns to the province to adequately offset the opportunity costs of protection in other areas. That said, other environmental concerns related to the oil sands, such as greenhouse gas emissions, water usage, pollution, and eventual reclamation would still need to be addressed.

The design element with the greatest effect on opportunity cost was the ecological representation target. We found that the relationship between the representation target and opportunity cost was nonlinear. This is again a consequence of the highly skewed distribution of resource values among planning units. Low-value planning units are most common and were preferentially selected for achieving representation targets. But when the representation target was high enough that high-value planning units had to be utilized, the cost of the reserve system increased more rapidly. Land managers could use this relationship (e.g., the point of inflection) to help guide their choice of representation target, particularly when the alternative is to use a politically-derived target lacking an objective basis.

Intactness can be added as a design feature with minimal incremental cost. This is because infrastructure development has been most prevalent in regions of higher resource value (Figs. S2 and S3). Consequently, instead of a trade-off situation, the selection of planning units with low resource value tends to favour the selection of planning units with lower levels of fragmentation.

When clumping was added as a design feature, using the boundary-length penalty, the model had less flexibility in utilizing low-cost planning units and the opportunity cost of the reserve system increased. At maximal levels of clumping the cost was approximately double that of scenarios that did not include clumping. Although this cost is not inconsequential, neither are the ecological benefits. Planning units that are part of a large contiguous protected area are much more likely to maintain ecological integrity and contribute to the long-term persistence of species than planning units that exist as isolated islands in a matrix of industrial development [28], [29], [30], [44]. Put another way, while isolated planning units may be “cheap” in terms of opportunity cost, they represent poor value in terms of ecological benefits per dollar spent.

Our attempt to add connectivity using major river corridors was largely unsuccessful. The only way to avoid gaps in our simulated corridors was to force all the relevant planning units into the model. This was effective in generating contiguous corridors, which we assumed was necessary for meaningful connectivity, but resulted in an opportunity cost that was more than ten times higher than a comparable scenario without corridors. Moreover, most of the added corridors did not serve their intended purpose because they did not link reserves together. This does not imply that Alberta's major river corridors do not merit protection, but it does suggest that a fine-filter approach may be more appropriate for these features than the township-scale coarse-filter approach used in our study. Other approaches will be needed for achieving connectivity among reserves [45], [46].

Although our modeling approach was not effective for linking reserves using corridors we were able to achieve substantial connectivity within the reserve system itself through the application of clumping. When the boundary length penalty was maximal the reserve system was dominated by three large contiguous reserves, two of which adjoined large existing protected areas (Fig. 4). The intrinsic connectivity provided by such a clumped design is likely to be superior to the connectivity of a dispersed design linked by long-distance corridors, particularly when the distances between reserves are large, as they are in our study area [47], [48], [49]. It is also worth noting that the establishment of corridors is also likely to result in additional economic opportunity costs, depending on the restrictions put in place.

When planning was conducted at the regional scale opportunity costs increased by up to four times over provincial-scale planning. This was because all targets had to be achieved locally, even if lower-cost planning units capable of achieving the same targets were available elsewhere. The regionally planned reserves were also inferior in ecological terms to the designs generated at the provincial scale: many of the selected planning units were located adjacent to private agricultural lands, where negative edge effects are likely to be greatest [44]. In addition, connectivity was poorer because the reserves were more dispersed, and in many cases separated by large distances.

Based on our findings one could reasonably expect that opportunity costs would be even lower if planning was conducted at the national scale, because even greater flexibility would be available for finding low-cost design solutions. However, the assumption of habitat substitutability that underlies the coarse-filter approach has limits. If ecosystem delineation and representation do not occur at a reasonable scale the prospects for achieving a meaningful conservation outcome are diminished [50], [51]. Put another way, a coarse filter that is too coarse is not a useful tool.

Jurisdiction must also be taken into account because in Canada responsibility for the management of provincial lands and resources rests with provincial governments, not the federal government. In addition, the opportunity costs (and benefits) of establishing reserves on public lands are largely borne by citizens of a given province, and less so by the nation as a whole. Allocation decisions based on a national-scale optimization of costs and benefits may not be supported provincially if the opportunity costs of protection are perceived as high in a local context [51], [52]. We conclude that provincial-scale planning may be most appropriate, though input from national-scale analyses could and should provide input into the planning process [42], [51], [53].

As with any modeling study, our findings must be considered in light of underlying assumptions and simplifications. One of these assumptions is that opportunity costs have been adequately quantified using our estimates of the NPV of petroleum and forestry resources. A concern is that our estimates of NPV, however well grounded by government data, may not be predictive of opportunity costs in the future because of unforeseen events. For example, a pine beetle attack might greatly diminish the value of forest resources in one region while other parts of the province rise in value because of new resource discoveries, technological advances, or changes in resource prices. Given the impossibility of addressing all such contingencies, the opportunity costs used in our study should not be considered accurate projections of the future but elements of plausible and meaningful modeling scenarios that are useful in the context of strategic decision making.

A related concern is that not all costs and benefits have been included in our analysis. Though it is clear that reserves provide societal benefits beyond the conservation of biodiversity, estimating of the equivalent dollar value of these benefits and their distribution across space was beyond the scope of this study. If these benefits were accounted for the net opportunity cost of protection would be lower than reported here [54]. Furthermore, the establishment of new reserves does not imply the simple idling of industrial capacity, but a reallocation to other parts of the landscape. This also serves to reduce real opportunity costs. The implication is that our findings regarding the trade-offs between economic opportunity costs and conservation objectives represent a worst-case scenario (in terms of cost).

Opportunity costs related to other resources can be discounted because petroleum and forestry account for more than 99% of resource revenues in our study area [55]. The costs of compensating companies for the loss of tenure rights in prospective reserves were not included in our analysis because the applicable rates have not been established. Assuming that compensation is linked to the loss of future revenues, and hence correlated with NPV, the relative ranking of planning units and their selection by Marxan should not be materially affected.

Interpretation of our findings should also take into account the small number of ecological design elements in our study. Though boreal landscapes have fewer species and less variability than many other biomes [56], it is unlikely that a simple coarse-filter design will address the needs of all species. As with other coarse-filter applications, a complementary fine-filter approach will be required to achieve comprehensive protection [15], [24].

In conclusion, our case study demonstrates that optimal resource allocation can be usefully employed for coarse-filter conservation initiatives, even when conservation targets are not well defined. The hybrid approach we used provides land managers with efficiently designed reserve options and a clear understanding of the economic trade-offs inherent in decisions concerning conservation design. This provides an objective basis for strategic decision making, thereby helping address the “implementation crisis” that plagues conservation science [8]. There also exists a potential for conservation gains if, as in our case, it can be demonstrated that the economic consequences of protection are less than expected. This is most likely to occur when the spatial variance of opportunity cost is high.

## Supporting Information

Figure S1 **The Natural Subregions of Alberta.** Note that grassland and parkland subregions were largely excluded from the analysis because they contain little public land (see Fig. 1).
(TIF)

Figure S2 **Net present value of petroleum and forestry resources, by township.**
(TIF)

Figure S3 **Density of linear features, by township.**
(TIF)

Figure S4 **Corridors used in the Marxan analysis, based on major rivers.**
(TIF)

Figure S5 **The seven planning regions designated under the Alberta Land-use Framework.**
(TIF)

Table S1 **Sources of data used in the Marxan analysis.**
(DOC)
