# Supplementary figures and images for: Achieving Conservation when Opportunity Costs Are High: Optimizing Reserve Design in Alberta's Oil Sands Region
Source: PLoS One. 2011 Aug 17;6(8):e23254. doi: 10.1371/journal.pone.0023254 (PMC3157348; doi:10.1371/journal.pone.0023254)

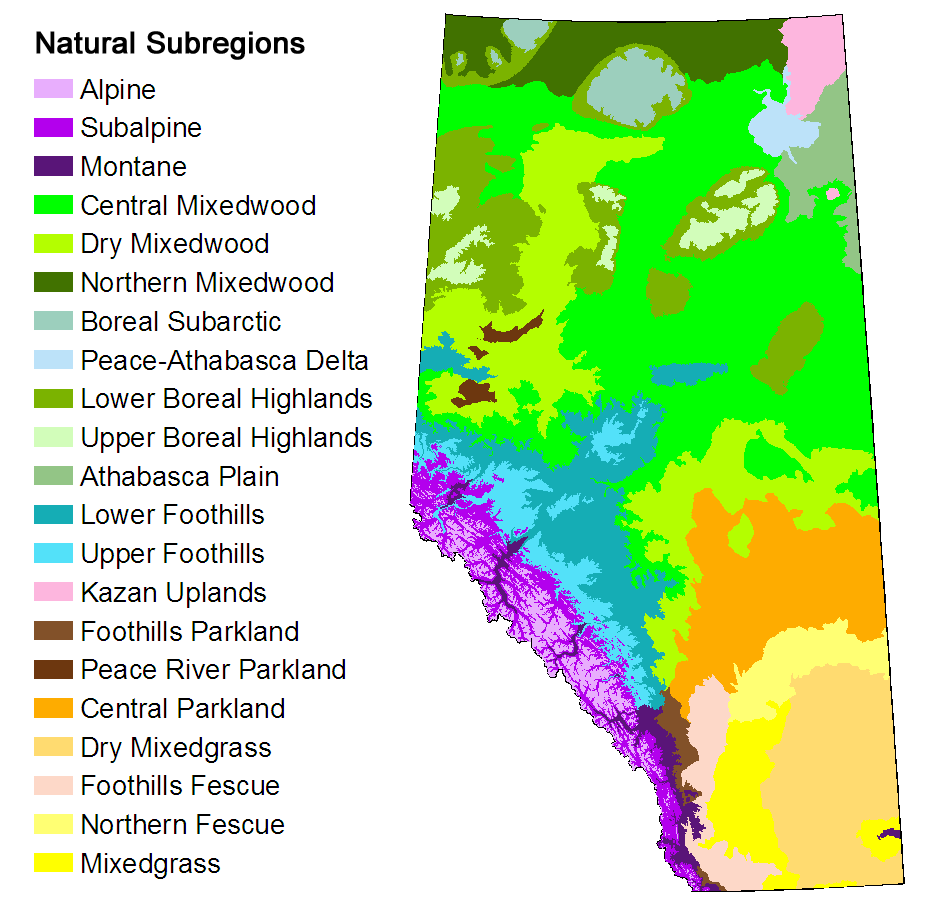

Supplement: Figure S1 — The Natural Subregions of Alberta. Note that grassland and parkland subregions were largely excluded from the analysis because they contain little public land (see Fig. 1). (TIF) [file pone.0023254.s001.tif]

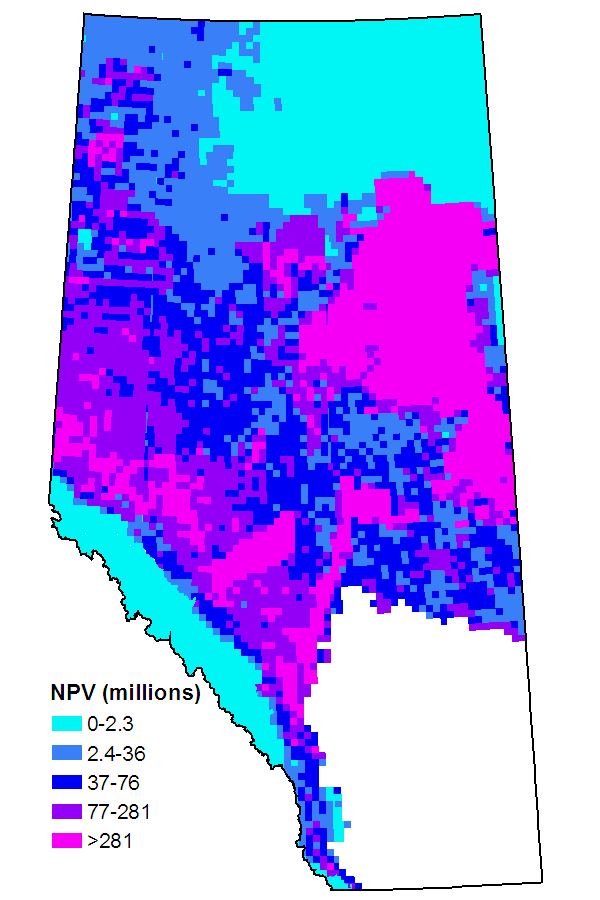

Supplement: Figure S2 — Net present value of petroleum and forestry resources, by township. (TIF) [file pone.0023254.s002.tif]

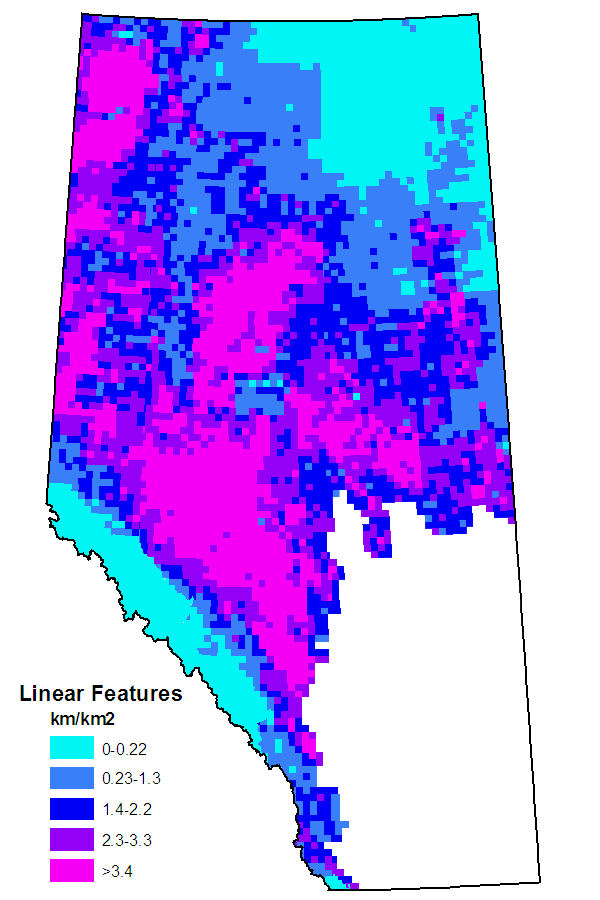

Supplement: Figure S3 — Density of linear features, by township. (TIF) [file pone.0023254.s003.tif]

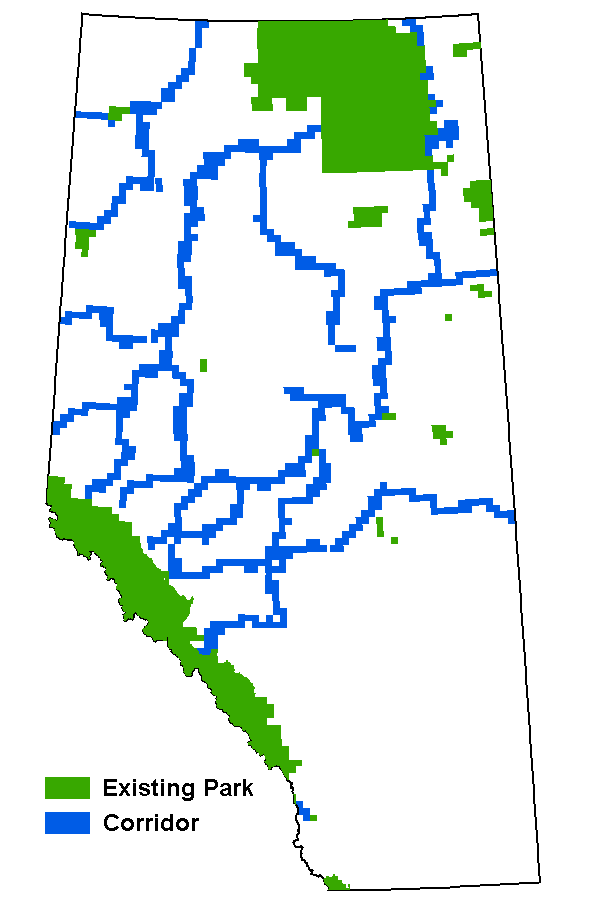

Supplement: Figure S4 — Corridors used in the Marxan analysis, based on major rivers. (TIF) [file pone.0023254.s004.tif]

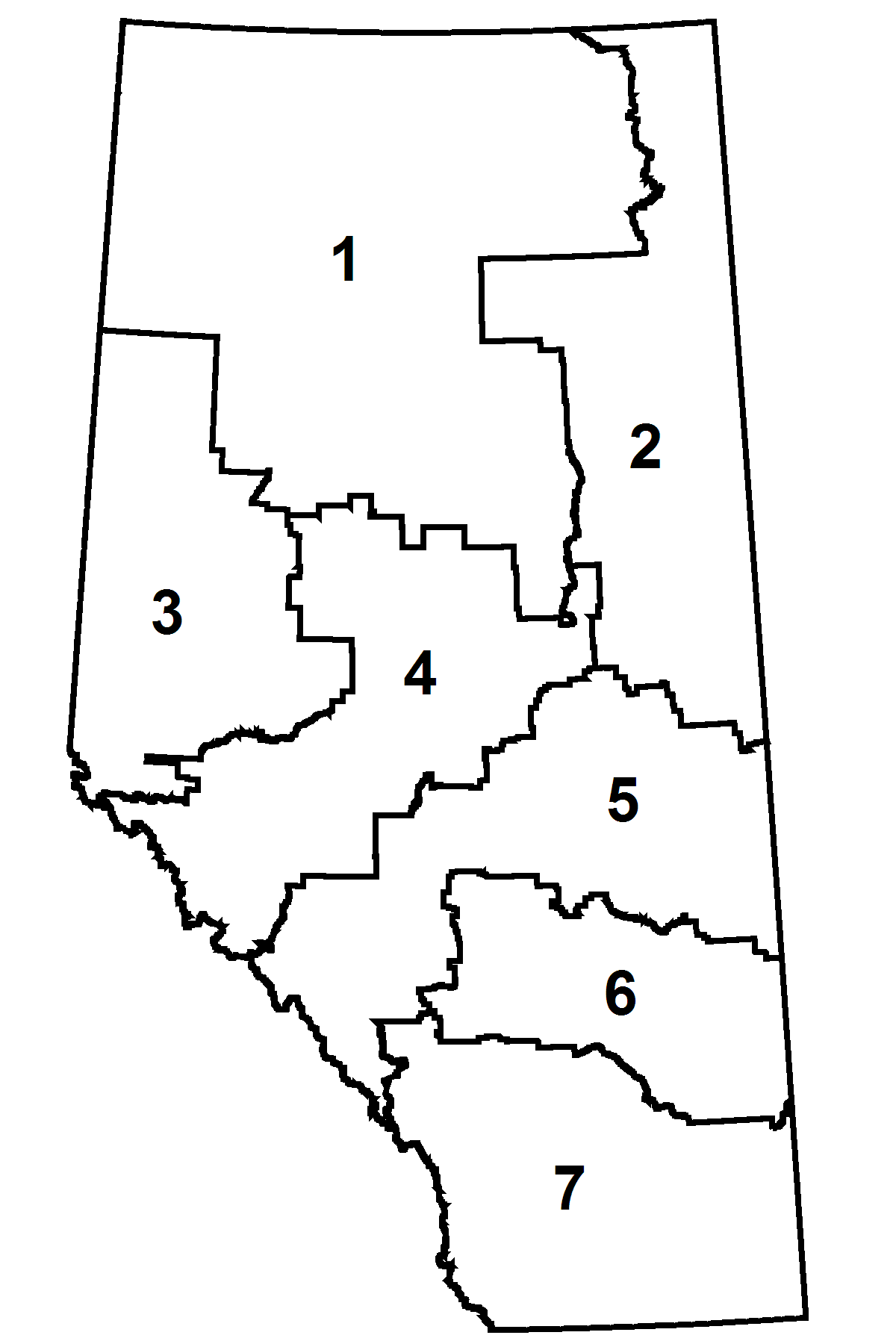

Supplement: Figure S5 — The seven planning regions designated under the Alberta Land-use Framework. (TIF) [file pone.0023254.s005.tif]
