# Supplementary material for: Achieving Conservation when Opportunity Costs Are High: Optimizing Reserve Design in Alberta's Oil Sands Region
Source: PLoS One. 2011 Aug 17;6(8):e23254. doi: 10.1371/journal.pone.0023254 (PMC3157348; doi:10.1371/journal.pone.0023254)
Supplement: Table S1 — Sources of data used in the Marxan analysis. (DOC) [file pone.0023254.s006.doc]

**Table S1.** Sources of data used in the Marxan analysis.

| **Data Layer** | **Source** |
| --- | --- |
| Existing protected areas | Alberta Base Features dataset, Government of Alberta, 2005-2009 [1] |
| Land ownership | Alberta Sustainable Resource Development, 2010 |
| Natural Subregions of Alberta | Alberta Sustainable Resource Development, 2005 [2] |
| Phase 3 forest inventory | Alberta Sustainable Resource Development, 2000 |
| Linear features | Alberta Base Features dataset, Government of Alberta, 2006 [1] |
| Townships | Alberta Base Features dataset, Government of Alberta, 2003 [1] |
| Rivers | Alberta Base Features dataset, Government of Alberta, 2003 [1] |
| Land-use Framework regional planning boundaries | Alberta Base Features dataset, Government of Alberta, 2009 [1] |

Notes:

[1] Available at www.altalis.com/prod_base_bound.html

[2] Available at http://tpr.alberta.ca/parks/heritageinfocentre/naturalregions/default.aspx
